# [Expression of Concern] Demethylation of microRNA-142 induced by demethylation agents plays a suppressive role in osteosarcoma cells

**DOI:** 10.3892/ol.2026.15837

**Published:** 2026-09-01

**Authors:** Muliang Ding, Jianzhong Hu, Jiangdong Ni, Zhonghui Zheng, Deye Song, Junjie Wang

Oncol Lett 9: 2261–2267, 2015; DOI: 10.3892/ol.2015.3036

Following the publication of this paper, it was drawn to the Editor's attention by a concerned reader that the data panels shown in Fig. 3C and E on p. 2264 portraying the results of cell invasion assay experiments contained three pairs of overlapping sections of data, such that data which were intended to show the results of differently performed experiments had apparently been derived from the same original sources.

The authors have been contacted by the Editorial Office to offer an explanation for the apparent anomalies in the presentation of the data in this paper, and we are awaiting their response. Owing to the fact that the Editorial Office has been made aware of potential issues surrounding the scientific integrity of this paper, we are issuing an Expression of Concern to notify readers of this potential problem while the Editorial Office continues to investigate this matter further.

